# Case Report: Gastrointestinal-onset hereditary transthyretin amyloidosis with cardiomyopathy and equivocal ^99m^Tc-PYP scintigraphy: diagnosis by florbetapir PET-guided skeletal muscle biopsy and genetic testing

**DOI:** 10.3389/fcvm.2026.1928392

**Published:** 2026-10-14

**Authors:** Shida Cai, Shisheng Miao, Guohui Yan, Junyu Chen, Rui Li, Qun Fan, Guoqiang Chen, Hong Zhang, Tingting Bai, Yuhan Ye, Suifeng Liu

**Affiliations:** 1 Department of Cardiology, Zhongshan Hospital Xiamen University, Xiamen, China; 2 Department of Echocardiography, Zhongshan Hospital Xiamen University, Xiamen, China; 3 Department of Nuclear Medicine, Zhongshan Hospital Xiamen University, Xiamen, China; 4 Department of Pathology, Zhongshan Hospital Xiamen University, Xiamen, China

**Keywords:** cardiac amyloidosis, equivocal ^99m^Tc-PYP, florbetapir PET/CT, hereditary transthyretin amyloidosis, p.Thr79Lys

## Abstract

**Background:**

Hereditary transthyretin amyloidosis (ATTRv) is a multisystem disease driven by pathogenic transthyretin *(TTR)* variants. Gastrointestinal presentations may precede cardiomyopathy, leading to diagnostic delay.

**Methods and results:**

A 52-year-old man presented with refractory watery diarrhoea, weight loss, orthostatic hypotension, bradycardia, and distal sensory symptoms; electromyography confirmed carpal tunnel syndrome. Cardiac imaging strongly suggested cardiac amyloidosis, while monoclonal protein studies were negative. Rectal biopsy confirmed amyloid. ^99m^Technetium-pyrophosphate (^99m^Tc-PYP) scintigraphy was equivocal with a Perugini score of 1. Because endomyocardial biopsy was declined, ^1^⁸F-florbetapir positron-emission tomography/computed tomography (^1^⁸F-florbetapir PET/CT) demonstrated myocardial and right gluteal muscle uptake, directing a gluteal biopsy that yielded amyloid confirmation. Whole exome sequencing identified a heterozygous *TTR* c.236C > A (p.Thr79Lys; historically p.Thr59Lys) variant. Liquid chromatography-tandem mass spectrometry (LC-MS/MS) of the amyloid-laden tissue identified TTR (88.7% coverage) with two mutant peptides confirming this substitution. Tafamidis 61 mg daily and dapagliflozin 10 mg daily were initiated. After 3 months, diarrhoea, dyspnoea, and fatigue improved; NYHA class improved to II and left-ventricular ejection fraction (LVEF) increased from 36% to 49%, accompanied by a reduction in N-terminal pro-B-type natriuretic peptide (NT-proBNP) (from 887 to 408 pg/mL) and high-sensitivity troponin T (hs-TnT) (from 23.9 to 12.1 ng/L).

**Conclusion:**

Refractory diarrhoea with orthostatic hypotension and carpal tunnel syndrome should raise suspicion for ATTRv. Equivocal bone scintigraphy does not exclude hereditary ATTR cardiomyopathy, and amyloid binding PET can guide safer extracardiac biopsy. Integrating multimodality imaging, histology, and genetics shortens diagnostic delay in this now treatable disease.

## Introduction

Hereditary transthyretin amyloidosis (ATTRv) exhibits an autosomal dominant inheritance pattern, arising from destabilising *TTR* variants that promote tetramer dissociation, monomer misfolding, and extracellular amyloid deposition in multiple organs, most notably the heart, peripheral nerves, and gastrointestinal tract (1–3). Cardiac involvement is a major determinant of prognosis, and delayed diagnosis impedes access to disease-modifying therapy (1, 3, 4). Emerging tools such as amyloid-binding PET may help resolve equivocal bone scintigraphy findings.

Although ATTRv is classically associated with mixed cardiomyopathic and neuropathic phenotypes, gastrointestinal and autonomic symptoms may dominate early in the disease course and divert attention toward more common gastrointestinal disorders (5–8). This problem is becoming more clinically important as the therapeutic landscape expands and earlier recognition is increasingly linked to better outcomes (3, 4, 9). Herein, we report a gastrointestinal-predominant presentation of ATTRv with cardiomyopathy in which the diagnostic pathway was complicated by equivocal ^99m^Tc-PYP scintigraphy. The final diagnosis was achieved through multimodality imaging, direct detection of the mutant peptide by mass spectrometry, and genetic testing, highlighting a practical approach to this diagnostic challenge.

## Methods and results

### Clinical presentation and imaging findings

A 52-year-old man presented with a 1-year history of persistent watery diarrhoea (3–4 episodes daily), accompanied by acid reflux, upper abdominal discomfort, and numbness in the distal extremities. Previous gastroenterological investigations, including upper gastrointestinal endoscopy, had led to diagnoses of bile reflux gastritis and a colonic lesion treated by endoscopic submucosal dissection. Histopathological examination of the resected lesion showed a sessile serrated lesion with dysplasia. His diarrhoea remained refractory to symptomatic treatment. During the preceding 6 months, he also developed progressive exertional dyspnoea, fatigue, and unintentional weight loss of 10 kg. His medical history included gout and nephrolithiasis. There was no known family history of amyloidosis.

On examination, his blood pressure was 80/46 mmHg and heart rate was 52 beats/min, with no other specific cardiopulmonary or abdominal abnormalities. Baseline NT-proBNP was 887 pg/mL, hs-TnT was 23.9 ng/L, creatinine was 1.3 mg/dL, and estimated glomerular filtration rate (eGFR) was 62.7 mL/min/1.73 m^2^.

Given the bradycardia, hypotension, progressive dyspnoea, and unexplained systemic symptoms, transthoracic echocardiography was performed. This showed symmetrically increased left ventricular wall thickness (interventricular septal of 14.1 mm), reduced LVEF (36%), and grade II diastolic dysfunction. Two-dimensional speckle-tracking analysis demonstrated relative apical sparing on longitudinal strain imaging (Figures 1A,B), raising strong suspicion for cardiac amyloidosis. Cardiac magnetic resonance (CMR) imaging reinforced this impression, showing diffuse circumferential left ventricular wall thickening, markedly elevated native myocardial T1 values, extracellular volume values ranging from 49.85% to 52.47%, and diffuse subendocardial late gadolinium enhancement (LGE) affecting the left ventricle and interatrial septum (Figure 1C), findings highly suggestive of cardiac amyloidosis (10).

**Figure 1:**
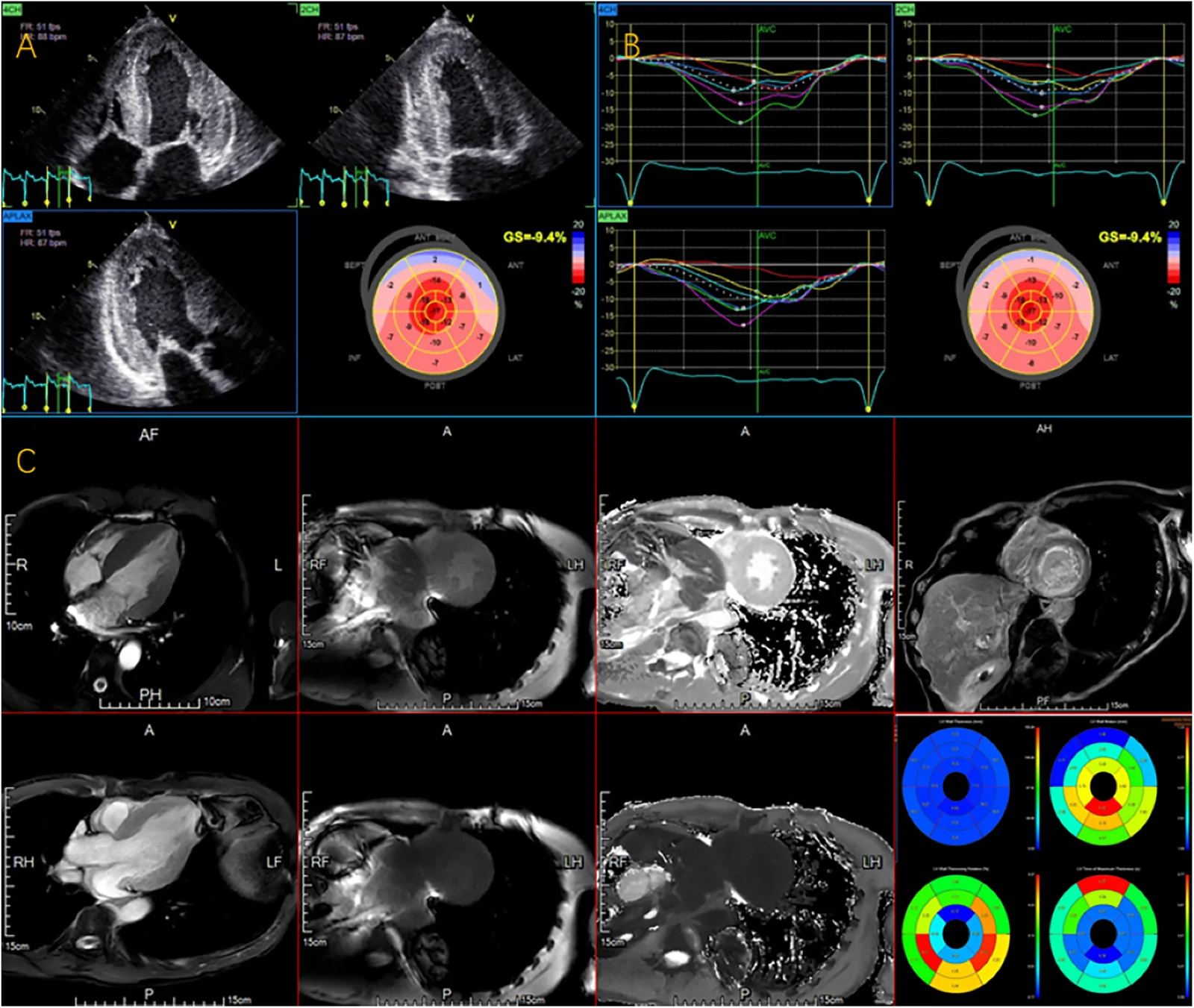
Cardiac imaging findings. **(A)** Two-dimensional echocardiography with global longitudinal strain bullseye visualisation; **(B)** Regional strain curves and bullseye plot of left ventricular myocardial deformation; **(C)** Cardiac magnetic resonance images demonstrating diffuse circumferential left ventricular wall thickening, markedly elevated native T1 values, and diffuse subendocardial late gadolinium enhancement (LGE) involving the left ventricle and interatrial septum (including T1 mapping, post-contrast, and LGE sequences).

In the evaluation of unexplained left ventricular hypertrophy, Fabry disease was also considered because of mildly reduced *α*-galactosidase A activity (1.70 μmol/L/h; reference range >2.0 μmol/L/h). However, subsequent *GLA* sequencing revealed no pathogenic variant, making classic Fabry disease very unlikely, although plasma lyso-Gb3 was not measured (11).

### Biopsy and histological confirmation

Aetiological evaluation was then undertaken. No monoclonal gammopathy was detected on serum/urine immunofixation or serum free light-chain testing**,** effectively ruling out AL amyloidosis. ^99m^Tc-PYP scintigraphy showed planar grade 1 myocardial uptake with a heart-to-contralateral lung ratio >1.42; overall, the result was interpreted as equivocal rather than diagnostic for transthyretin cardiac amyloidosis (12, 13).

Given the patient's impaired cardiac function and the risks of sedation and bowel preparation, comprehensive gastrointestinal endoscopy was not performed; instead, a low-risk rectal biopsy was carried out and confirmed amyloid deposition (Congo red positive with apple-green birefringence under polarized light) (Figures 2A,B). The patient declined endomyocardial biopsy. As the rectal biopsy specimen yielded insufficient tissue for definitive amyloid subtyping, ^18^F-florbetapir PET/CT was undertaken as an adjunctive imaging study to map the distribution of suspected amyloid deposition and identify a practical extracardiac biopsy target. This showed tracer uptake in the myocardium (maximum standardized uptake value SUV_max_ 2.7) and throughout the skeletal musculature (e.g., right gluteal muscle SUV_max_ 1.7), with a myocardial-to-blood pool SUV_max_ ratio of 4.5 (Figure 3). Guided by these findings, biopsy of the right gluteal muscle was performed and demonstrated amyloid deposition on Congo red staining (Figures 2C,D).

**Figure 2:**
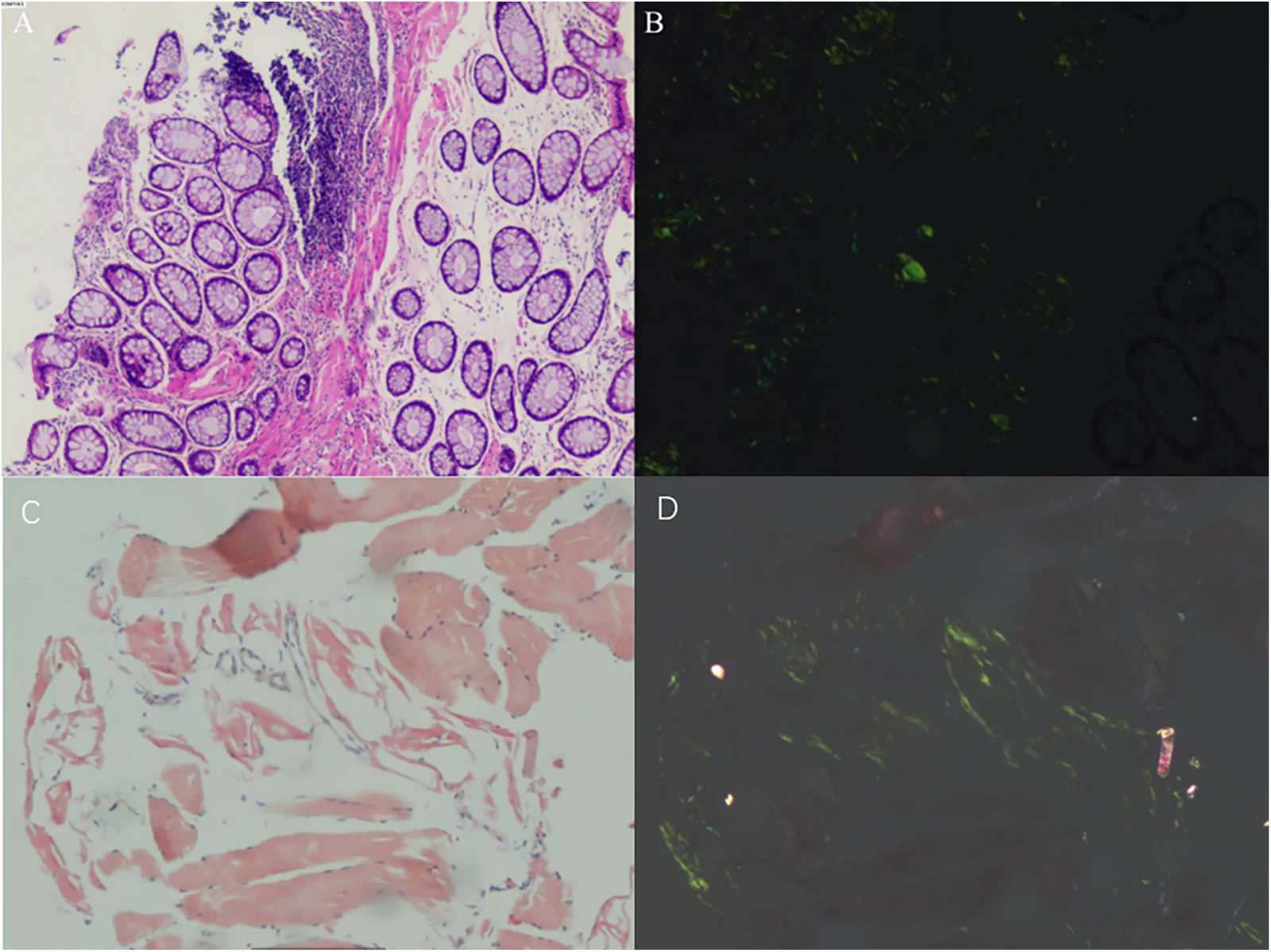
Histological findings from rectal biopsy (**A** and **B**). Congo red staining demonstrating amyloid deposition, **(A)** bright field; **(B)** apple-green birefringence (polarised light). Histological findings from right gluteal muscle biopsy (**C** and **D**). Congo red staining confirming amyloid deposition **(C)** bright field; **(D)** apple-green birefringence (polarised light).

**Figure 3:**
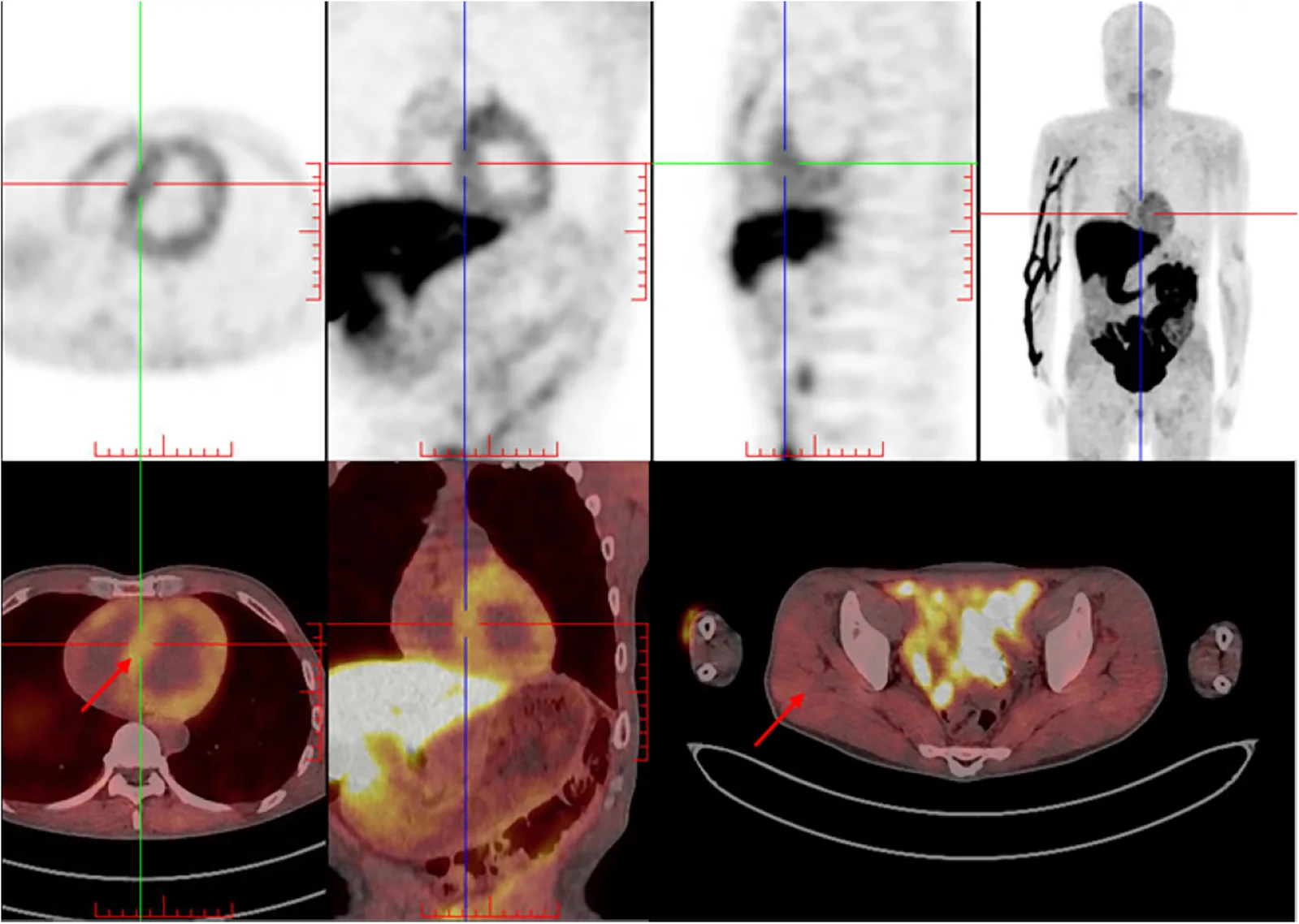
^18^F-florbetapir PET/CT imaging. Whole-body maximum intensity projection image showing tracer uptake in the myocardium (arrow in the left lower panel) and throughout the skeletal musculature, including the right gluteal muscle (arrow in the right lower panel).

### Genetic and proteomic confirmation

Whole-exome sequencing identified a heterozygous missense *TTR* variant, c.236C > A (p.Thr79Lys, historically designated Thr59Lys) (Figure 4A) (14–17). Although detailed genetic counselling was provided, the patient's asymptomatic adult son declined cascade genetic testing and long-term cardiac surveillance. To further confirm the presence of the mutant TTR protein in the amyloid deposits, LC-MS/MS was carried out on the amyloid-laden gluteal muscle tissue. This analysis identified TTR with 88.7% sequence coverage (12 unique peptides) (Figure 4B). A peptide spanning residues 69–90 (TSESGELHGLKTEEEFVEGIYK) was detected, which carries a lysine instead of threonine at position 79 (Figure 4C), directly demonstrating the presence of the variant protein in the amyloid deposits. Together with the genetic findings, these results confirmed the diagnosis of hereditary transthyretin amyloidosis with cardiac involvement.

**Figure 4:**
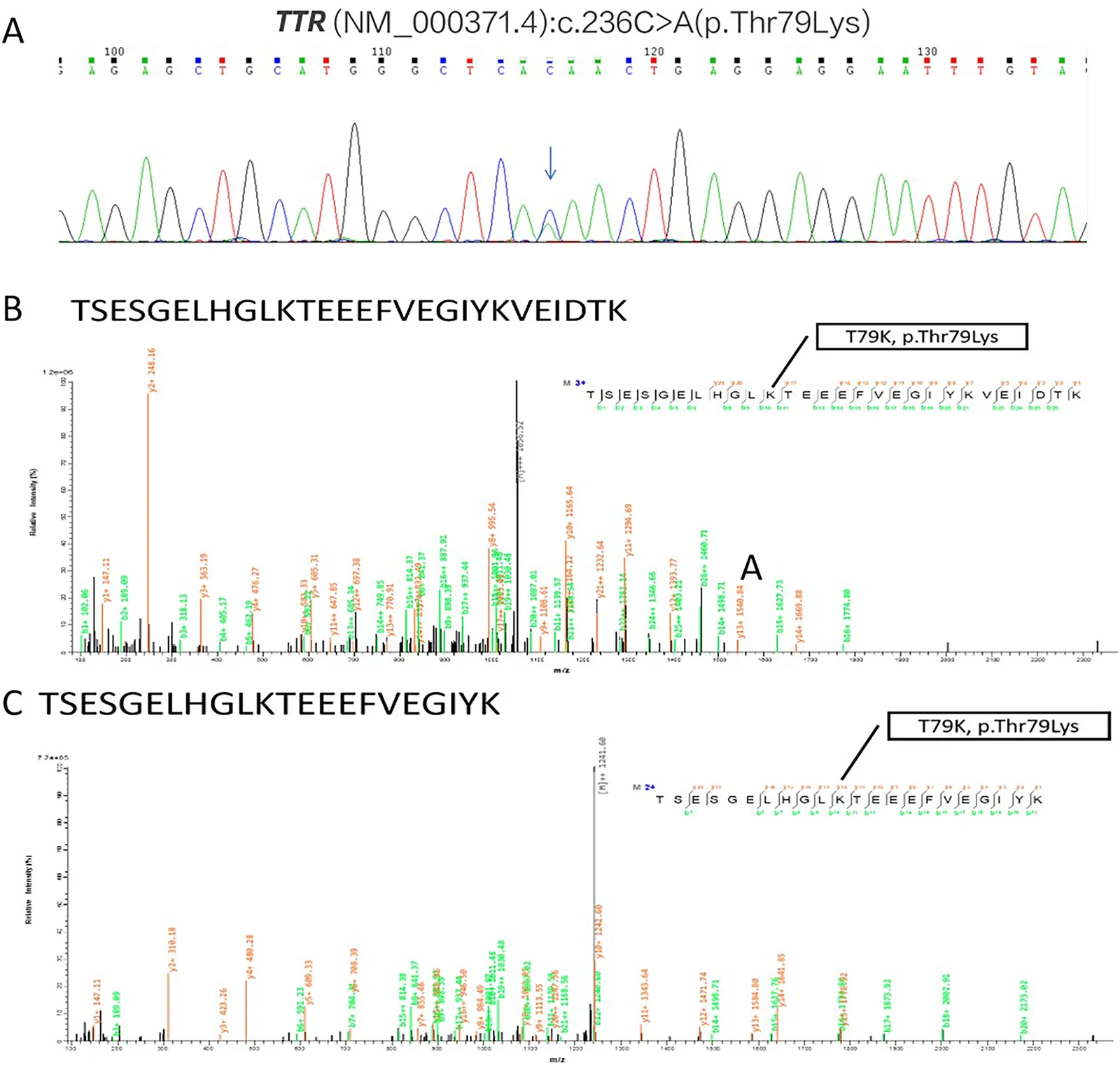
Sanger sequencing chromatogram of the TTR gene. The electropherogram displays the heterozygous c.236C > A (p.Thr79Lys) variant (arrow), confirming whole-exome sequencing findings **(A)** Proteomic confirmation of TTR variant in amyloid-laden tissue (**B** and **C**). Peptide mapping of two distinct mutant TTR peptides harbouring the p.Thr79Lys substitution, identified from amyloid-laden gluteal muscle tissue.

### Treatment and follow-up

Tafamidis 61 mg once daily and dapagliflozin 10 mg once daily were continued throughout the 3-month follow-up period. While additional guideline-directed medical therapy (GDMT) was considered, beta-blockers were avoided given bradycardia and hypotension, and sacubitril/valsartan (ARNI), vericiguat and mineralocorticoid receptor antagonists (MRAs) were not initiated due to persistent hypotension. At the 3-month follow-up visit, blood pressure was maintained at 100/60 mmHg and heart rate was 60 beats/min. Repeat laboratory testing yielded the following results: NT-proBNP 408 pg/mL, hs-TnT 12.1 ng/L, creatinine 1.27 mg/dL, eGFR 64.5 mL/min/1.73 m^2^. After three months of treatment, the patient exhibited substantial clinical improvement: diarrhoea frequency decreased from 3 to 4 episodes per day to 1–2 episodes per day, and exertional dyspnoea and fatigue improved. NYHA functional class improved from III to II. Follow-up echocardiography showed an increase in LVEF from 36% to 49%. Despite these objective and symptomatic improvements, the patient remained concerned about long-term disease progression and lifelong medication. A comprehensive chronological timeline of the patient's entire clinical course, from initial symptom onset to the 3-month follow-up, is detailed in Supplementary Table S1 and visually summarised in Supplementary Figure S1.

## Discussion

This case highlights how ATTRv can first enter the differential diagnosis through the gastrointestinal clinic rather than the cardiology clinic. Chronic diarrhoea, weight loss, orthostatic hypotension, and distal sensory symptoms can reflect a combination of enteric/autonomic neuropathy and direct gastrointestinal amyloid deposition (5–8). Because these manifestations are non-specific, they are easily attributed to functional or inflammatory gastrointestinal disorders, especially when cardiac symptoms are absent or still evolving. In the present case, the coexistence of refractory diarrhoea, orthostatic hypotension, neuropathic symptoms, and later cardiac abnormalities was the key signal that the disease was multisystem rather than isolated gastrointestinal pathology.

The case also illustrates an important limitation of the current non-biopsy diagnostic pathway for transthyretin cardiac amyloidosis. Echocardiography and cardiac magnetic resonance generated a highly coherent cardiac amyloid phenotype, and monoclonal protein testing substantially lowered the likelihood of immunoglobulin light chain (AL) cardiac amyloidosis. However, ^99m^Tc-PYP scintigraphy was only grade 1. This finding should be interpreted cautiously: grade 0 or 1 uptake does not exclude ATTR,particularly in hereditary disease and in selected *TTR* genotypes with reduced tracer sensitivity (12, 13, 18, 19). Therefore, an equivocal bone scintigraphy result should not terminate the evaluation when the pre-test probability remains high.

The right gluteal muscle was selected as the biopsy target because ^1^⁸F-florbetapir PET/CT demonstrated increased tracer uptake at this site (SUV_max_ 1.7). This site offered favourable accessibility and safety. Conventional biopsy approaches, including abdominal subcutaneous fat sampling, were considered. Although prior rectal biopsy confirmed systemic amyloidosis, the tissue sample was insufficient for definitive TTR subtyping, and rectal biopsy carried inherent bleeding risk in amyloid-infiltrated, fragile vasculature. Abdominal subcutaneous fat biopsy was expected to have low diagnostic yield in this clinical context. This gluteal muscle biopsy represented an independent sampling procedure rather than an extension of the prior rectal biopsy, with lower anticipated procedural risk compared with endomyocardial biopsy.

A clinically instructive aspect of this report is the use of florbetapir PET/CT to confirm cardiac involvement in the setting of equivocal bone scintigraphy. Bone scintigraphy is highly specific for ATTR cardiac amyloidosis but cannot detect AL cardiac amyloidosis and has reduced sensitivity in certain hereditary *TTR* genotypes (20, 21). In contrast, amyloid-binding tracers—such as ^11^C-PiB, ^18^F-florbetapir, ^18^F-florbetaben, and the novel pan-amyloid agent ^124^I-evuzamitide—bind directly to *β*-sheet amyloid fibrils, enabling the detection of both ATTR and AL cardiac amyloidosis, and exhibit differential uptake patterns (AL > ATTR > controls) that can aid in subtype differentiation (22). For instance, ^18^F-florbetaben differentiates AL from ATTR cardiac amyloidosis with 90%–93% accuracy using myocardial/blood SUV_max_, while ^124^I-evuzamitide demonstrates 94% sensitivity and 86% specificity in the Phase III REVEAL study (22–24). Beyond diagnosis, ^18^F-florbetapir myocardial burden may decrease following plasma cell-directed therapy in AL amyloidosis, correlating with NT-proBNP, suggesting a role in monitoring treatment response (25).

In our case, modest myocardial florbetapir uptake (SUV_max_ 2.7) was consistent with its lower affinity for ATTR compared with AL, while diffuse skeletal muscle involvement, including the right gluteal muscle (SUV_max_ 1.7), guided the diagnostic biopsy. These findings highlight that the potential value of amyloid-binding PET lies less in stand-alone diagnosis than in problem-solving: mapping disease distribution, supporting biopsy planning, and preserving diagnostic momentum in difficult cases (20, 21, 26). This may be particularly valuable when bone scintigraphy yields indeterminate findings, the preferred biopsy site is not feasible, or clinicians wish to avoid a higher-risk invasive procedure. Nevertheless, florbetapir PET/CT is not recommended as a first-line investigation; it is associated with high cost and limited accessibility, and prospective comparisons with alternative strategies are required before wider clinical adoption.

Genetic confirmation was essential. The detected variant is designated p.Thr79Lys using current sequence-based nomenclature, whereas older reports often use p.Thr59Lys because of historical mature-protein numbering (14–17). Recognising this nomenclature shift is important for accurate literature review and variant interpretation. Of note, p.Thr79Lys is documented in only a small number of older case reports, and robust genotype-phenotype data are lacking for this rare variant; therefore, the gastrointestinal-predominant phenotype seen here should not be overinterpreted. The absence of a known family history did not exclude ATTRv, because penetrance may be incomplete and relatives may remain undiagnosed.

Long-term management of ATTRv cardiomyopathy requires multidisciplinary follow-up. Real-world data support the acceptable tolerability of tafamidis for ATTRv cardiomyopathy (27), and dapagliflozin is generally well tolerated in heart failure treatment (28). No drug-related adverse events were noted during the 3-month follow-up in this patient. Beyond safety, the favourable cardiac response observed at reassessment may be multifactorial. Specifically, the LVEF improvement observed at 3 months likely reflects a combination of tafamidis, dapagliflozin, decongestion-related volume optimisation, and the resolution of reversible functional components. Objectively, the clinical improvement was accompanied by reductions in NT-proBNP and hs-TnT, alongside stable renal function. Given the hereditary nature of the condition and the confirmed genotype, genetic counselling was offered and cascade genetic testing was recommended for first-degree relatives (29). Nevertheless, the patient's asymptomatic adult son declined genetic screening and long-term cardiac surveillance. The long-term prognosis for this patient remains uncertain and depends on sustained therapeutic response, cardiac function, nutritional status, and progression of peripheral neuropathy.

This retrospective case report has several important limitations. First, this is a single-patient observation, precluding generalisable conclusions. Second, the follow-up duration was limited to three months; the observed LVEF improvement cannot be definitively attributed to tafamidis therapy, and long-term sustained benefit remains to be confirmed. Additionally, serial global longitudinal strain (GLS) data were not available at this early time point, precluding a more sensitive assessment of subclinical myocardial recovery. Third, no head-to-head comparison was performed between florbetapir-PET-guided skeletal-muscle biopsy and conventional biopsy strategies. Fourth, formal autonomic function testing, tilt-table testing, and heart rate variability analysis were not performed. Fifth, plasma lyso-Gb3 was not assayed, precluding complete exclusion of Fabry disease. Despite these limitations, the case generates a testable hypothesis: in patients with suspected ATTRv and non-diagnostic bone scintigraphy, amyloid-binding PET may have a useful role in selecting a lower-risk extracardiac biopsy target. This question warrants prospective study.

## Conclusion

Persistent unexplained diarrhoea should raise clinical suspicion for hereditary transthyretin amyloidosis when accompanied by weight loss, orthostatic hypotension, neuropathic symptoms, or unexplained cardiac abnormalities, even in the setting of equivocal bone scintigraphy. A stepwise strategy integrating multimodality cardiac imaging, extracardiac histology, and genetic-proteomic testing can secure the diagnosis even when endomyocardial biopsy is declined or contraindicated due to high risk. In carefully selected patients, florbetapir PET/CT may help identify a safer extracardiac biopsy target, but its cost, limited accessibility, and exact clinical role require further study.

## Data Availability

The data supporting the findings of this study are included in the article/supplementary material, further inquiries can be directed to the corresponding author/s.
