# Supplementary material for: Case Report: Gastrointestinal-onset hereditary transthyretin amyloidosis with cardiomyopathy and equivocal 99mTc-PYP scintigraphy: diagnosis by florbetapir PET-guided skeletal muscle biopsy and genetic testing
Source: Front Cardiovasc Med. 2026 Oct 14;13:1928392. doi: 10.3389/fcvm.2026.1928392 (PMC13713733; doi:10.3389/fcvm.2026.1928392)
Supplement: Supplementary file 1 [file Table1.docx]

**Table S1.** Approximate diagnostic timeline.

| Time point |  | Event |
| --- | --- | --- |
| Month 0 |  | Onset of watery diarrhoea |
| Month 6 |  | Development of exertional dyspnoea, fatigue, weight loss, distal sensory symptoms |
| Month 12 |  | First presentation; NYHA III, BP 80/46 mmHg, HR 52 beats/min |
| Month 12.2 |  | Echocardiography and CMR suggestive of cardiac amyloidosis |
| Month 12.3 |  | ^99m^Tc-PYP scintigraphy equivocal (grade 1); monoclonal protein studies negative |
| Month 12.5 |  | Rectal biopsy confirms amyloid |
| Month 13 |  | ^18^F-florbetapir PET/CT shows myocardial and skeletal muscle uptake |
| Month 13.2 |  | Right gluteal muscle biopsy confirms amyloid |
| Month 13.4 |  | WES identifies *TTR* c.236C>A (p.Thr79Lys); LC-MS/MS confirms mutant TTR peptide |
| Month 13.5 |  | Tafamidis 61 mg daily and dapagliflozin 10 mg daily initiated |
| Month 16.5 |  | 3-month follow-up: NYHA II, diarrhoea improved, LVEF 36%→49% |

NYHA, New York Heart Association; BP, blood pressure; HR, heart rate; 99mTc-PYP, technetium-99m pyrophosphate; PET/CT, positron emission tomography/computed tomography; WES, whole-exome sequencing; TTR, transthyretin; LC-MS/MS, liquid chromatography-tandem mass spectrometry; LVEF, left ventricular ejection fraction.

¹ Month 0 is defined as symptom onset of watery diarrhoea. Decimal values indicate fractions of one month.
